# Disseminated paracoccidioidomycosis with skull and mandible involvement in a heart transplant recipient

**DOI:** 10.1590/0037-8682-0110-2022

**Published:** 2022-06-06

**Authors:** Fernanda Veloso Pereira, Katariny Parreira de Oliveira Alves, Albina Messias de Almeida Milani Altemani, Fabiano Reis

**Affiliations:** 1 Universidade Estadual de Campinas, Faculdade de Ciências Médicas, Departamento de Radiologia, Campinas, SP, Brasil.; 2 Universidade Estadual de Campinas, Faculdade de Ciências Médicas, Departamento de Patologia, Campinas, SP, Brasil.

**FIGURE 1:**
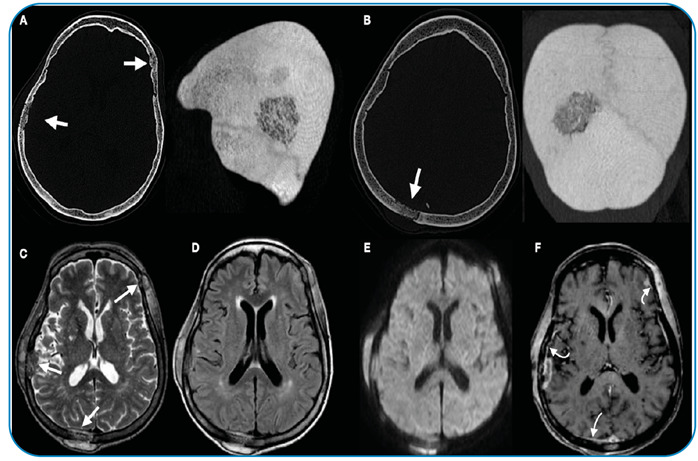
CT of the brain showing the permeative osteolytic pattern involving the left frontal and right temporal bones **(A)** and parietal bones **(B)**. The permeative osteolytic pattern of the lesions is better demonstrated with MIP reconstruction. MRI of the brain showed lesions with a solid component and low signal on T2 WI **(C)** and on FLAIR WI **(D)**, restricted diffusion on DWI **(E)**, and heterogeneous enhancement on T1-WI after gadolinium in addition to pachymeningeal enhancement adjacent to the lesions (**white curved arrow in F**).

**FIGURE 2:**
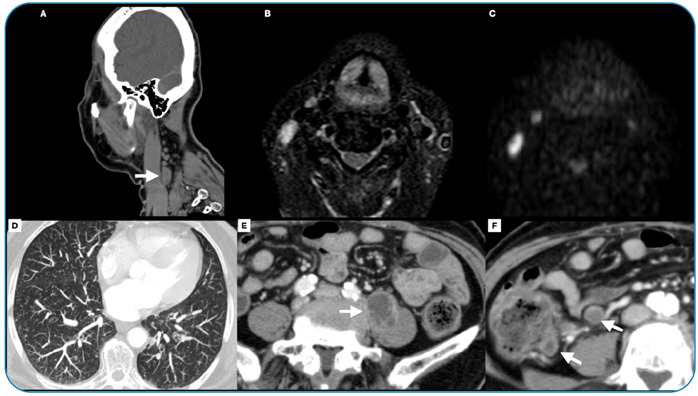
CT and MRI of the neck showing lymph node enlargement in the right level V **(white arrow in A, B, and C)** and a CT of the chest showing the presence of numerous small diffuse peripheral nodules **(D)**. A CT of the abdomen also revealed an abscess in the left iliopsoas muscle **(E)** and necrotic lymph nodes **(F)**, demonstrated with a white arrow.

**FIGURE 3:**
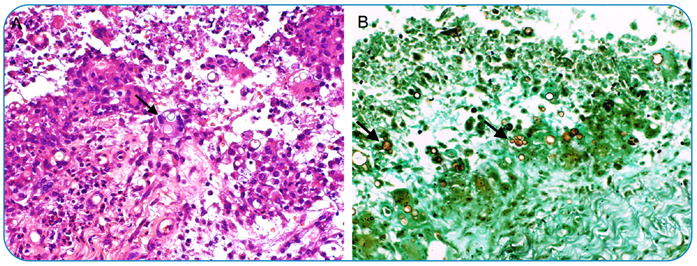
**(A)** Paracoccidioidomycosis shows a prominent granulomatous inflammatory infiltrate with multinucleated giant cells containing fungal organisms (black arrow). Hematoxylin-eosin, 200x. **(B)** The budding yeast form of the fungus shows a classic "steering wheel" morphology (black arrows). Grocott's methenamine stain, 200x.

Paracoccidioidomycosis (PCM) is the most common systemic mycosis in South America, particularly in Brazil. The respiratory airways are the main route of entry and usually spread through the vascular and lymphatic systems, affecting any part of the body. Herein, we report the case of a 59-year-old woman, recipient of a heart transplant, who was being treated with mycophenolate, cyclosporine, and tacrolimus and presented intense pulsatile holocranial headache associated with nodules on the scalp and trismus. Brain computed tomography (CT) revealed skull lesions (Figures 1A and B ). Magnetic resonance imaging (MRI) of the brain was performed (Figures 1C-F). CT of the neck revealed lymph node enlargement and osteolytic lesions in the mandible. Chest CT revealed numerous small peripheral lung nodules, and abdominal CT detected an abscess in the left iliopsoas muscle (Figure 2). The fungus showed a "steering wheel" morphology detected using Grocott's methenamine staining (Figure 3).

In solid organ transplantations, chronic PCM has been described mainly in kidney transplants. Almeida et al.^1^ described nine cases of PCM after kidney transplantation and one case in a liver transplant recipient. PCM in immunocompromised heart transplant patients has not been reported.

PCM can develop after hematological dissemination from an active pulmonary infection or through the reactivation of a latent focus in the central nervous system after immunosuppression^2^.

The patient responded well to treatment with voriconazole followed by cotrimoxazole. This treatment schedule was based on standard antifungal therapy^3^.
